# Robust Adaptive Control for a Class of Uncertain Nonlinear Systems with Time-Varying Delay

**DOI:** 10.1155/2013/963986

**Published:** 2013-06-17

**Authors:** Ruliang Wang, Jie Li, Shanshan Zhang, Dongmei Gao, Huanlong Sun

**Affiliations:** ^1^Computer and Information Engineering College, Guangxi Teachers Education University, Nanning 530023, China; ^2^Yantai Nanshan Vocational Technology School, Yantai, China; ^3^School of Mathematical Sciences, Guangxi Teachers Education University, Nanning 530023, China; ^4^Department of Basic Courses, Haikou College of Economics, Haikou, China

## Abstract

We present adaptive neural control design for a class of perturbed nonlinear MIMO time-varying delay systems in a block-triangular form. Based on a neural controller, it is obtained by constructing a quadratic-type Lyapunov-Krasovskii functional, which efficiently avoids the controller singularity. The proposed control guarantees that all closed-loop signals remain bounded, while the output tracking error dynamics converge to a neighborhood of the desired trajectories. The simulation results demonstrate the effectiveness of the proposed control scheme.

## 1. Introduction

In the practical control process, control system is usually required to meet the stability and the corresponding performance index, which affects the system stability factors mainly including the uncertainties and time delays. On the study of the uncertain time-delay many scholars have achieved valuable fruits [1, 2]. Paper [3] has analyzed and designed the optimal *H*_*∞*_ feedback controller by the LMI method. In recent decades, the delay nonlinear systems with neural network research have received extensive attention [4–20]. Paper [4] has solved the problem of chaotic synchronization phenomenon by the neural network method. In [5–11], the study of the nonlinear continuous system and discrete nonlinear system is based on adaptive neural network control. The tracking and stabilization problem of nonlinear systems has been studied by neural network backstepping method [12, 13]. In [14], neural network control has been applied to a piece of triangle structure of multiple-input multiple-output nonlinear time-delay system, in which a dynamic system neural network is used mainly for unknown function approximation and separation. In multiple input and multiple output nonlinear system, [15] presents a new adaptive neural network controller design method but does not consider with external disturbance and time-varying delay. In [18], the problem of the adaptive neural networks control for a class of nonlinear state-delay systems with unknown virtual control coefficients is considered. In [19], a control scheme combined with backstepping, radial basis function (RBF) neural networks and adaptive control are proposed for the stabilization of nonlinear system with input and state delay.

This paper mainly aims at studying the simultaneous presence of uncertainties and time-varying delay MIMO nonlinear system. By defining the new quadratic Lyapunov-Krasovskii functionals, it has analyzed and designed the adaptive neural network controller by neural network approximation method in [15, 16].

## 2. Description of the Problem

Let us consider the following block-triangular structure with the disturbance of nonlinear MIMO systems with time-varying delays:

(1) x˙j,ij=fj,ij(x−j,ij)+gj,ij(x−j,ij)xj,ij+1+hj,ij(x−τj,ij)+ωj,ij(t),x˙j,mj=fj,mj(X,u−j−1)+gj,mj(X,u−j−1)uj+hj,mj(Xτ)+ωj,mj(t),yj=xj,1, j=1,…,n,  ij=1,…,mj−1,

where ${\overset{-}{x}}_{j,i_{j}}={\left[x_{j,1},\ldots ,x_{j,i_{j}}\right]}^{T}\in R^{i_{j}}$ are the state variable for the *i*_*j*_ differential equations of the *j*th subsystem; *X* = [*x*_1_^*T*^,…, *x*_*n*_^*T*^]^*T*^, where *x*_*j*_ = [*x*_*j*,1_,…, *x*_*j*,*m*_*j*__]^*T*^ ∈ *R* ^*m*_*j*_^ are the state vector of the *j*th subsystem; *x*_*τ*_*j*,*i*_*j*___ = *x*_*j*,*i*_*j*__(*t* − *τ*_*j*,*i*_*j*_(*t*)_), where *τ*_*j*,*i*_*j*_(*t*)_ are unknown time-varying delay of the states, and $|\tau_{j,i_{j}}(t)|\leq \tau_{j,i_{j}},\;|{\dot{\tau }}_{j,i_{j}}(t)|\leq \tau_{1}<1,\;\;\tau_{0}=\max\left\{\tau_{j,i_{j}}|1\leq j\leq n,\;1\leq i_{j}\leq m_{j}\right\}$, ${\overset{-}{x}}_{\tau_{j,i_{j}}}={\left[x_{\tau_{j,1}},\ldots ,x_{\tau_{j,i_{j}}}\right]}^{T}$, *X*_*τ*_ = [*x*_*τ*_1,1__,…, *x*_*τ*_1,*n*_1___,…, *x*_*τ*_*n*,1__,…, *x*_*τ*_*n*,*m*_*n*___]^*T*^, the output *y* = [*y*_1_,…, *y*_*n*_]^*T*^ ∈ *R* ^*n*^; ${\overset{-}{u}}_{j}={\left[u_{1},\ldots ,u_{j}\right]}^{T}$ are input vector of the *j*th subsystem; *f*_*j*,*i*_*j*__(·), *g*_*j*,*i*_*j*__(·), and *h*_*j*,*i*_*j*__(·) are unknown smooth nonlinear function. *ω*_*j*,*i*_*j*__(*t*) is the disturbance input and |*ω*_*j*,*i*_*j*__(*t*)|≤*d*_*j*,*i*_*j*__ < 1. Let *x*_*j*,*i*_*j*__(*t*) = *β*_*j*,*i*_*j*__(*t*), with *t* ∈ [−*τ*_0_, 0]; assume *β*_*j*,*i*_*j*__(*t*) is smooth and bounded.

We make the following assumptions for the system (1).

**Assumption 1**

The desired trajectories *y*_*dj*_,  *j* = 1,2,…, *n*, have the *n*th derivation and the derivation is continuous and bounded.

**Assumption 2**

We use *g*_*j*,*i*_*j*__(·) to represent some given function. There exist constant *g*_*j*0_ and unknown smooth functions ${\overset{-}{g}}_{j,i_{j}}(\cdot )$, such that $0<g_{j0}\leq |g_{j,i_{j}}(\cdot )|\leq {\overset{-}{g}}_{j,i_{j}}(\cdot )<\infty$. Without loss of generality, we further assume that *g*_*j*,*i*_*j*__(·) > *g*_*j*0_ > 0.

**Lemma 3 (see [16])**

There exists smooth positive function *ψ*_*j*_(*η*_*j*_) : *R* ^*m*_*j*_^ → *R*  (*j* = 1,2,…, *n*) with *ψ*_*j*_(0) = 0 for all continuous functions *h*(*η*_1_,…, *η*_*n*_) : *R* ^*m*_1_^ × ⋯×*R* ^*m*_*n*_^ → *R* with *h*(0,…, 0) = 0, where *η*_*j*_ ∈ *R* ^*m*_*j*_^  (*j* − 1,2,…, *n*, *m*_*j*_ > 0), such that |*h*(*η*_1_,…, *η*_*n*_)|≤∑_*j*=1_^*n*^ *ψ*_*j*_(*η*_*j*_).

**Lemma 4 (see [14])**

On any normal number *ξ* > 0 and random variable *l* ∈ *R* have lim⁡_*l*→0_⁡tan*h* ^2^(*l*/*ξ*)/*l* = 0.

In this paper, the following radial basis function neural network is used to approximate unknown continuous function (in [13] once had been put forward):

(2) $$\begin{array}{c} f\left(Z\right)=W^{T}S\left(Z\right)+\theta \left(Z\right),\;\left|\theta \left(Z\right)\right|\leq \varepsilon ,\;\;\left(\varepsilon >0\right), \end{array}$$

where the input vector *Z* ∈ *Ω*_*Z*_ ⊂ *R* ^*n*^; *W* = [*w*_1_, *w*_2_,…, *w*_*l*_]^*T*^ is the weight vector; the number of neural network node *l* > 1 and *S*(*Z*) = [*s*_1_(*Z*), *s*_2_(*Z*),…, *s*_*l*_(*Z*)]^*T*^, where *s*_*i*_ = exp⁡[−(*Z* − *μ*_*i*_)^*T*^(*Z* − *μ*_*i*_)/*ϕ*_*i*_^2^], *i* = 1,2,…, *l*, *μ*_*i*_ = [*μ*_*i*1_, *μ*_*i*2_,…, *μ*_*in*_]^*T*^ is the center of the receptive field, and *ϕ*_*i*_ is the width of the Gaussian function.

## 3. Adaptive Neural Network Controller Design

In this section, we will introduce a novel adaptive NN control design procedure. There are *m*_*j*_ design steps in the design procedure for the *j*th subsystem. In each step, the unknown nonlinear function ${\overset{-}{f}}_{j,i_{j}}(Z_{j,i_{j}})$ will be approximated by a radial neural network approximation function. Define an unknown constant as

(3) $$\begin{array}{c} \alpha_{j}=\frac{1}{g_{j0}}\max\left\{{||W_{j,i_{j}}||}^{2}:1\leq i_{j}\leq m_{j}\right\}, \end{array}$$

where the constant *g*_*j*0_ is defined as in Assumption 2; function ${\overset{-}{f}}_{j,i_{j}}$ and vector *Z*_*j*,*i*_*j*__ will be specified in each step. Furthermore, for *j* = 1,2,…, *n* and *i*_*j*_ = 1,2,…, *m*_*j*−1_, choose the virtual control laws as follows:

(4) λj,ij=−(kj,ij+1)zj,ij−12aj,ij2α^jzj,ijST(Zj,ij)S(Zj,ij),

where *k*_*j*,*i*_*j*__ > 0 and *a*_*j*,*i*_*j*__ > 0 are design parameters, ${\hat{\alpha }}_{j}$ represent the estimation of the unknown constant *α*_*j*_, and *S*(·) is the basis function vector, and define the variables *z*_*j*,*i*_*j*__ as follows:

(5) $$\begin{array}{c} z_{j,i_{j}}=x_{j,i_{j}}-\lambda_{j,i_{j-1}},\;\;z_{j,1}=x_{j,1}-y_{dj}, \end{array}$$

for *j* = 1,…, *n*, *i*_*j*_ = 2,…, *m*_*j*_. Choose the adaptive laws ${\dot{\hat{\alpha }}}_{j}$ as follows:

(6) $$\begin{array}{c} {\dot{\hat{\alpha }}}_{j}=\sum_{i_{j}=1}^{m_{j}}\frac{r_{j}}{2a_{j,i_{j}}^{2}}z_{j,i_{j}}^{2}S^{T}\left(Z_{j,i_{j}}\right)S\left(Z_{j,i_{j}}\right)-b_{j}{\hat{\alpha }}_{j}, \end{array}$$

where *r*_*j*_ > 0 and *b*_*j*_ > 0 are design parameters.

*Step* *j* · 1  (1 ≤ *j* ≤ *n*). For the first differential equation of the *j*th subsystem, choose the Lyapunov function candidate

(7) $$\begin{array}{c} V_{z_{j,1}}=\frac{1}{2}z_{j,1}^{2}+\frac{g_{j0}}{2r_{j}}{\tilde{\alpha }}^{2}, \end{array}$$

where $z_{j,1}=x_{j,1}-y_{dj},\;\;\tilde{\alpha }=\alpha_{j}-{\hat{\alpha }}_{j}$. Taking the time derivative of *V*_*z*_*j*,1__, we obtain

(8) V˙zj,1=zj,1(fj,1+gj,1λj,1−y˙dj+hj,1(x−τj,1)+ωj,1(t))+zj,1gj,1zj,2−gj0rjα~jα^˙j.

With Lemma 3, existence of positive function *Q*_*j*,*l*_^*j*,*i*_*j*_^(*x*_*τ*_*j*,*l*__)  *l* = 1,2,…, *i*_*j*_, such that

(9) $$\begin{array}{c} \left|h_{j,i_{j}}\left({\overset{-}{x}}_{\tau_{j,i_{j}}}\right)\right|\leq \sum_{l=1}^{i_{j}}Q_{j,l}^{j,i_{j}}\left(x_{\tau_{j,l}}\right). \end{array}$$

Then, we have

(10) $$\begin{array}{c} z_{j,1}h_{j,1}\left({\overset{-}{x}}_{\tau_{j,1}}\right)\leq \left|z_{j,1}\right|Q_{j,1}^{j,1}\leq \frac{1}{2}z_{j,1}^{2}+\frac{1}{2}{\left[Q_{j,1}^{j,1}\left(x_{\tau_{j,1}}\right)\right]}^{2}. \end{array}$$

Substituting (10) into (8) yields

(11) V˙zj,1≤zj,1(fj,1+gj,1λj,1−y˙dj+12zj,1+ωj,1(t))+zj,1gj,1zj,2+12[Qj,1j,1(xτj,1)]2−gj0rjα~jα^˙j.

To overcome the time-varying delay terms of (11), consider the following Lyapunov-Krasovskii functional:

(12) $$\begin{array}{c} V_{j,1}=V_{z_{j,1}}+V_{u_{j,1}}, \end{array}$$

where

(13) $$\begin{array}{c} V_{u_{j,1}}=\int_{t-\tau_{j,1}(t)}^{t}\frac{1}{2\left(1-\tau_{1}\right)}{\left[Q_{j,1}^{j,1}\left(x_{j,1}\left(s\right)\right)\right]}^{2}ds. \end{array}$$

Take the time derivative of *V*_*u*_*j*,1__:

(14) V˙uj,1≤12(1−τ1)[Qj,1j,1(xj,1(t))]2−12[Qj,1j,1(xj,1(t−τj,1(t)))]2;

from (11) and (14), one has

(15) V˙j,1≤zj,1(f−j,1(Zj,1)+gj,1λj,1+ωj,1(t))−gj0rjα~jα^˙j+zj,1gj,1zj,2+[1−2tanh2(zj,1ηj,1)]Uj,1,

where

(16) $$\begin{array}{c} Z_{j,1}={\left[x_{j,1},y_{dj},{\dot{y}}_{dj},{\hat{\alpha }}_{j}\right]}^{T},\\ U_{j,1}=\frac{1}{2\left(1-\tau_{1}\right)}{\left[Q_{j,1}^{j,1}\left(x_{j,1}\right)\right]}^{2},\\ {\overset{-}{f}}_{j,1}\left(Z_{j,1}\right)=f_{j,1}-{\dot{y}}_{dj}+\frac{1}{2}z_{j,1}+\frac{2}{z_{j,1}}\tan h^{2}\left(\frac{z_{j,1}}{\eta_{j,1}}\right)U_{j,1}, \end{array}$$

and *η*_*j*,1_ is a positive constant.

From Lemma 4, the function (1/*z*)tan*h* ^2^(*z*/*η*) is defined at *z* = 0 and can be approximated by a neural network. Therefor the function ${\overset{-}{f}}_{j,1}$ will be approximated by the NN *W*_*j*,1_^*T*^ *S*(*Z*_*j*,1_), such that, for given *ε*_*j*,1_ > 0,

(17) $$\begin{array}{c} {\overset{-}{f}}_{j,1}\left(Z_{j,1}\right)=W_{j,1}^{T}S\left(Z_{j,1}\right)+\theta_{j,1}\left(Z_{j,1}\right),\;\left|\theta_{j,1}\left(Z_{j,1}\right)\right|\leq \varepsilon_{j,1}, \end{array}$$

where *θ*_*j*,1_(*Z*_*j*,1_) is the approximation error. Furthermore, a straightforward calculation shows that

(18) zj,1f−j,1(Zj,1)≤12aj,12gj0zj,12αjST(Zj,1)S(Zj,1)+12aj,12+12gj0zj,12+12εj,12gj0−1.

In additions, from (6), we obtain that for any initial conditions ${\hat{\alpha }}_{j}(t_{0})\geq 0$, ${\hat{\alpha }}_{j}(t)>0$ for all *t* > *t*_0_. Therefor

(19) zj,1gj,1λj,1≤−gj02aj,12α^jzj,12ST(Zj,1)S(Zj,1)−(kj,1+1)gj0zj,12,

(20) $$\begin{array}{c} z_{j,1}\omega_{j,1}\left(t\right)\leq \frac{1}{2}g_{j0}z_{j,1}^{2}+\frac{1}{2}d_{j,1}^{2}g_{j0}^{-1}. \end{array}$$

Substituting (18)–(20) into (15) yields that

(21) V˙j,1≤kj,1gj0zj,12+12(aj,12+εj,12gj0−1+dj,12gj0−1)+gj0rjα~j(rj2aj,12zj,12ST(Zj,1)S(Zj,1)−α^˙j)+zj,1gj,1zj,2+[1−2tanh2(zj,1ηj,1)]Uj,1.  

*Step* *j* · *i*_*j*_  (*i*_*j*_ = 2,…, *m*_*j*_ − 1). Define the Lyapunov-Krasovskii functional as

(22) $$\begin{array}{c} V_{z_{j,i_{j}}}=\frac{1}{2}z_{j,i_{j}}^{2}; \end{array}$$

differentiating *V*_*z*_*j*,*i*_*j*___ yields

(23) V˙zj,ij=zj,ij(fj,ij+gj,ijxj,ij+1−λ˙j,ij−1   +hj,ij(x−τj,ij)+ωj,ij(t)).

From (10), we have

(24) $$\begin{array}{c} z_{j,i_{j}}h_{j,i_{j}}\leq \sum_{k=1}^{i_{j}}\left(\frac{1}{2}z_{j,i_{j}}^{2}+\frac{1}{2}{\left[Q_{j,k}^{j,i_{j}}\left(x_{\tau_{j,k}}\right)\right]}^{2}\right); \end{array}$$

${\dot{\lambda }}_{j,i_{j}-1}(Z_{j,i_{j}-1})$ can be expressed as

(25) λ˙j,ij−1=∑k=1ij−1∂λj,ij−1∂xj,k(fj,k+gj,kxj,k+1+ωj,k)+∑k=0ij−1∂λj,ij−1∂ydj(k)ydj(k+1)+∂λj,ij−1∂α^jα^˙j+∑k=1ij−1∂λj,ij−1∂xj,khj,k(x−τj,k).

Similar to (24), we can get

(26) −zj,ij∑k=1ij−1∂λj,ij−1∂xj,khj,k(x−τj,k)≤∑k=1ij−1‍ ∑l=1k12zj,ij2[∂λj,ij−1∂xj,k]2+∑k=1ij−1 ∑l=1k12[Qj,lj,k(xτj,l)]2.

Substituting (24)–(26) into (23) yields that

(27) V˙zj,ij≤zj,ij(fj,ij+gj,ijxj,ij+1+ωj,ij   −∑k=1ij−1∂λj,ij−1∂xj,k(fj,k+gj,kxj,k+1+ωj,k)   +∑k=1ij12zj,ij−∑k=0ij−1∂λj,ij−1∂ydj(k)ydj(k+1)   +∑k=1ij−1 ∑l=1k12zj,ij[∂λj,ij−1∂xj,k]2)−∂λj,ij−1∂α^jα^˙j+∑k=1ij−1 ∑l=1k12[Qj,lj,k(xτj,l)]2+∑k=1ij12[Qj,kj,ij(xτj,k)]2.

To overcome the delay terms in (27), let us consider the following Lyapunov-Krasovskii functional:

(28) $$\begin{array}{c} V_{j,i_{j}}=V_{z_{j,i_{j}}}+V_{u_{j,i_{j}}}, \end{array}$$

where

(29) Vuj,ij=∑k=1ij∫t−τj,kt12(1−τ1)[Qj,kj,ij(xj,k(s))]2ds+∑k=1ij ∑l=1k∫t−τj,lt12(1−τ1)[Qj,lj,k(xj,l(s))]2ds.

Differentiating *V*_*u*_*j*,*i*_*j*___ yields

(30) V˙uj,ij=∑k=1ij12(1−τ1)[Qj,kj,ij(xj,k(t))]2+∑k=1ij−1 ∑l=1k12(1−τ1)[Qj,lj,k(xj,l(t))]2−∑k=1ij12(1−τ1)[Qj,kj,ij(xτj,ij)]2(1−τ˙j,k)−∑k=1ij−1 ‍∑l=1k12(1−τ1)[Qj,lj,k(xτj,l)]2(1−τ˙j,l)≤Uj,ij−∑k=1ij12[Qj,kj,ij(xτj,ij)]2−∑k=1ij−1 ‍∑l=1k12[Qj,lj,k(xτj,l)]2≤zj,ij2zj,ijtanh2(zj,ijηj,ij)Uj,ij+[1−2tanh2(zj,ijηj,ij)]Uj,ij−∑k=1ij12[Qj,kj,ij(xτj,ij)]2−∑k=1ij−1 ∑l=1k12[Qj,lj,k(xτj,l)]2,

where

(31) Uj,ij=∑k=1ij12(1−τ1)[Qj,kj,ij(xj,k(t))]2+∑k=1ij−1 ∑l=1k12(1−τ1)[Qj,lj,k(xj,l(t))]2.

Then, combining (27) and (30) results in

(32) V˙j,ij≤zj,ij(φj,ij−∂λj,ij−1∂α^jα^˙j)+gj,ijzj,ijzj,ij+1+[1−2tanh2(zj,ijηj,ij)]Uj,ij+zj,ij(f−j,ij+gj,ijλj,ij+1+ωj,ij),

where

(33) f−j,ij=fj,ij−∑k=1ij−1∂λj,ij−1∂xj,k(fj,k+gj,kxj,k+1+ωj,k)−∑k=0ij−1∂λj,ij−1∂ydj(k)ydj(k+1)+∑k=1ij12zj,ij+∑k=1ij−1 ∑l=1k12zj,ij[∂λj,ij−1∂xj,k]2+2zj,ijtanh2(zj,ijηj,ij)Uj,ij−φj,ij.

The NN *W*_*j*,*i*_*j*__^*T*^ *S*(*Z*_*j*,*i*_*j*__) is used to approximate ${\overset{-}{f}}_{j,i_{j}}$ such that for given *ε*_*j*,*i*_*j*__ > 0 we have

(34) $$\begin{array}{c} {\overset{-}{f}}_{j,i_{j}}=W_{j,i_{j}}^{T}S\left(Z_{j,i_{j}}\right)+\theta_{j,i_{j}}\left(Z_{j,i_{j}}\right),\;\left|\theta_{j,i_{j}}\left(Z_{j,i_{j}}\right)\right|\leq \varepsilon_{j,i_{j}}, \end{array}$$

where *θ*_*j*,*i*_*j*__(*Z*_*j*,*i*_*j*__) represent the approximation error. Similar to (18) and (20), we have

(35) V˙j,ij≤−kj,ijgj0zj,ij2+12(aj,ij2+εj,ij2gj0−1+dj,ij2gj0−1)+gj0rjα~jrj2aj,ij2zj,ij2ST(Zj,ij)S(Zj,ij)+zj,ij(φj,ij−∂λj,ij−1∂α^jα^˙j)+gj,ijzj,ijzj,ij+1+[1−2tanh2(zj,ijηj,ij)]Uj,ij.

*Step* *j* · *m*_*j*_  (1 ≤ *j* ≤ *n*). In the final step of the *j*th subsystem to construct the actual control law *u*_*j*_, let us consider the following Lyapunov-Krasovskii function:

(36) $$\begin{array}{c} V_{j,i_{j}}=\frac{1}{2}z_{j,m_{j}}^{2}+V_{u_{j,m_{j}}}, \end{array}$$

where

(37) Vuj,mj=∑j=1n ∑k=1mj∫t−τj,kt12(1−τ1)[Qj,kj,mj(xj,k(s))]2ds+∑k=1mj−1 ∑l=1k∫t−τj,lt12(1−τ1)[Qj,lj,k(xj,l(s))]2ds

and *z*_*j*,*m*_*j*__ = *x*_*j*,*m*_*j*__ − *λ*_*j*,*m*_*j*_−1_. Similar to (32) we get

(38) V˙j,mj≤zj,mj(φj,mj−∂λj,mj−1∂α^jα^˙j)+[1−2tanh2(zj,mjηj,mj)]Uj,mj+zj,mj(f−j,mj+gj,mjuj+ωj,mj),

where ${\overset{-}{f}}_{j,m_{j}}(z_{j,m_{j}})$ can be defined by (33) with *i*_*j*_ = *m*_*j*_.

We use the NN *W*_*j*,*m*_*j*__^*T*^ *S*(*Z*_*j*,*m*_*j*__) to approximate ${\overset{-}{f}}_{j,m_{j}}$ such that, for given *ε*_*j*,*m*_*j*__ > 0, we have

(39) f−j,mj=Wj,mjTS(Zj,mj)+θj,mj(Zj,mj),|θj,mj(Zj,mj)|≤εj,mj,

where *θ*_*j*,*m*_*j*__(*Z*_*j*,*m*_*j*__) express the approximation error.

Choose the control law *u*_*j*_ as

(40) uj=−(kj,mj+1)zj,mj−12aj,mj2α^jzj,mjST(Zj,mj)S(Zj,mj).

Similar to (21) we have

(41) V˙j,mj≤12(aj,mj2+εj,mj2gj0−1+dj,mj2gj0−1)+gj0rjα~jrj2aj,mj2zj,mj2ST(Zj,mj)S(Zj,mj)−kj,mjgj0zj,mj2+zj,mj(φj,mj−∂λj,mj−1∂α^jα^˙j)+[1−2tanh2(zj,mjηj,mj)]Uj,mj.

Let *V*_*n*,*m*_*n*__ = ∑_*j*=1_^*n*^ ∑_*k*=1_^*m*_*j*_^ *V*_*j*,*k*_. Combining (21), (35), and (41) gives that

(42) V˙n,mn≤−∑j=1n ∑k=1mjkj,kgj0zj,k2+∑j=1n ∑k=1mj12(aj,k2+εj,k2gj0−1+dj,k2gj0−1)+∑j=1ngj0rjα~j(∑k=1mjrj2aj,k2zj,k2ST(Zj,k)S(Zj,k)−α^˙j)+∑j=1n ∑k=1mj[1−2tanh2(zj,kηj,k)]Uj,k+∑j=1n ∑k=2mjzj,k(φj,k−∂λj,k−1∂α^jα^˙j).

The control law design is thus completed.

## 4. Stability Analysis

Now, the main result in this paper can be presented as follows.

**Theorem 5**

Consider the nonlinear time-delay system (1) with the NN adaption law (6) and the control law (40) satisfying Assumptions 1–2. All the closed-loop trajectories can guarantee boundedness if the unknown function ${\overset{-}{f}}_{j,i_{j}}$ can be approximated by neural network and the approximating error *θ*_*j*,*i*_*j*__is boundedness.

**Proof**

Define functions *φ*_*j*,*k*_, such that

(43) $$\begin{array}{c} -\sum_{j=1}^{n}‍\;\sum_{k=2}^{m_{j}}z_{j,k}\left(\varphi_{j,k}-\frac{\partial \lambda_{j,k-1}}{\partial {\hat{\alpha }}_{j}}{\dot{\hat{\alpha }}}_{j}\right)\leq 0. \end{array}$$

Let 0 < *S* ^*T*^(·)*S*(·) < *L*, where *L* is the number of neural network weights.

From (6), we can get

(44) −∑k=2mjzj,k∂λj,k−1∂α^jα^˙j ≤∑k=2mjzj,k(bjα^j∂λj,k−1∂α^j      −∑l=1k−1∂λj,k−1∂α^jrj2aj,l2zj,l2ST(Zj,l)S(Zj,l))  +∑k=2mjzj,k(rjL2aj,k2zj,k2∑l=2k|zj,l∂λj,l−1∂α^j|).  

By choosing function *φ*_*j*,*k*_ as

(45) φj,k=−bjα^j∂λj,k−1∂α^j−rjL2aj,k2zj,k2∑l=2k|zj,l∂λj,l−1∂α^j|+  ∑l=1k−1∂λj,k−1∂α^jrj2aj,l2zj,l2ST(Zj,l)S(Zj,l),

(43) holds.

In a similar way, we can get

(46) ∑j=1ngj0rjα~j(∑k=1mjrj2aj,k2zj,k2ST(Zj,k)S(Zj,k)−α^˙j)  ≤∑j=1ngj0rj(−α~j2+αj2).

Now, choose the Lyapunov function as *V* = *V*_*n*,*m*_*n*__.

Combining (42)–(46) gives that

(47) V˙n,mn≤−∑j=1n‍ ∑k=1mjkj,kgj0zj,k2−∑j=1ngj0bjrjα~j2+∑j=1n ∑k=1mj[1−2tanh2(zj,kηj,k)]Uj,k+D,

where

(48) $$\begin{array}{c} D=\sum_{j=1}^{n}‍\;\sum_{k=1}^{m_{j}}\frac{1}{2}\left(a_{j,k}^{2}+\varepsilon_{j,k}^{2}g_{j0}^{-1}+d_{j,k}^{2}g_{j0}^{-1}\right)+\sum_{j=1}^{n}\frac{g_{j0}}{r_{j}}\alpha_{j}^{2} \end{array}$$

is a constant. Thus, by (47) the boundedness follows immediately from the same line used in the proof in [9–11].

## 5. Simulation Examples

In this section, we will give one example to demonstrate the effectiveness of the proposed method in this paper. Let us consider the following example:

(49) x˙1,1=−x1,1+(1+cos⁡2(x1,1))x1,2+xτ1,12+ω1,1(t),x˙1,2=x1,1x1,2+x2,1+x2,2+(1+0.5 cos⁡2(x2,2))u1 +  xτ1,2+ω1,2(t),x˙2,1=−x2,1+x2,2+xτ2,1+ω2,1(t),x˙2,2=(x1,2+x2,1)x2,2−x1,1u1+(2+sin2(u1))u2 +  xτ1,1xτ2,2+ω2,2(t),

where *x*_*τ*_*j*,*i*_*j*___ = *x*_*j*,*i*_*j*__(*t* − *τ*_*j*,*i*_*j*__), *j* = 1,2, *i*_*j*_ = 1,2.

And the time delays are chosen as

(50) $$\begin{array}{c} \tau_{1,1}=0.9+0.1\sin\left(t\right),\;\;\tau_{1,2}=1+0.5\sin\left(t\right),\\ \tau_{2,1}=0.4+0.1\cos\left(t\right),\;\;\tau_{2,2}=2+0.1\cos\left(t\right), \end{array}$$

given the reference output signals as *y*_*d*1_ = 0.5(sin(*t*) + sin(0.5*t*)), *y*_*d*2_ = 0.5sin(*t*) + sin(0.5*t*). The control law is given by (40). The NN adaptation law is given by (6). Choose the design parameters

(51) $$\begin{array}{c} k_{1,1}=k_{1,2}=k_{2,1}=k_{2,2}=20,\\ a_{1,1}=a_{1,2}=2,\;\;a_{2,1}=a_{2,2}=1,\\ r_{1}=r_{2}=400,\\ b_{1}=b_{2}=0.025. \end{array}$$

Take the external disturbance as

(52) $$\begin{array}{c} \omega_{1,1}\left(t\right)=\omega_{1,2}\left(t\right)=0.04\sin\left(2\pi t\right),\\ \omega_{2,1}\left(t\right)=\omega_{2,2}\left(t\right)=0.04\cos\left(2\pi t\right). \end{array}$$

**Figure 1:**
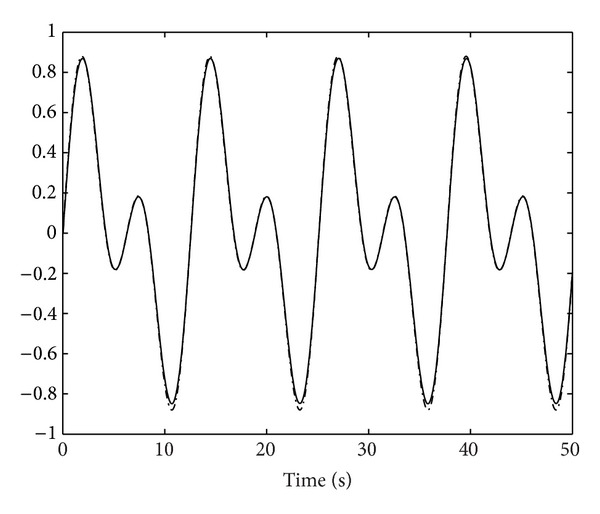
System output *y*_1_(*t*)(“−”) and the reference *y*_*d*1_(*t*)(“−·−”).

**Figure 2:**
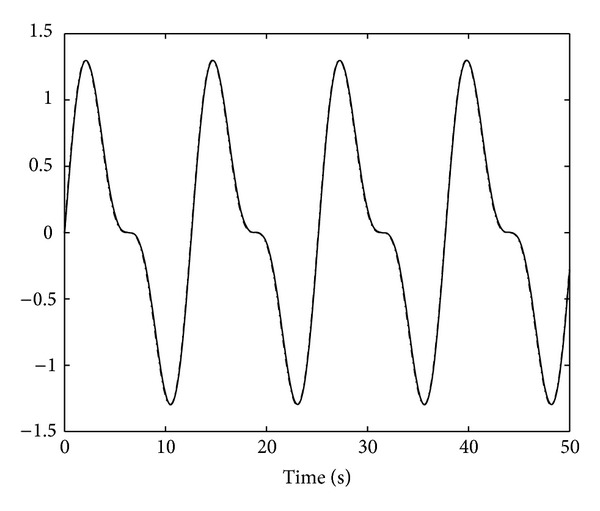
System output *y*_2_(*t*)(“−”) and the reference *y*_*d*2_(*t*) (“−·−”).

**Figure 3:**
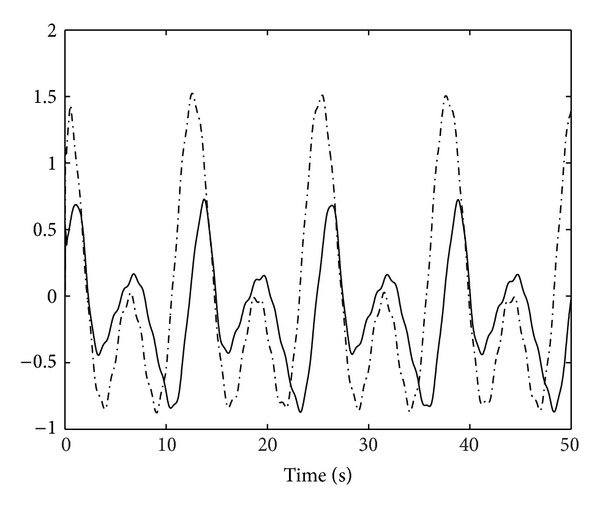
Responses of state variables *x*_1,2_ and *x*_2,2_.

**Figure 4:**
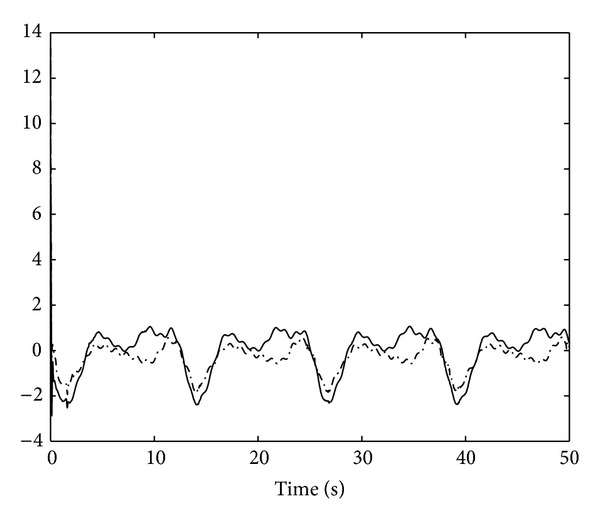
Control input signals *u*_1_ and *u*_2_.

**Figure 5:**
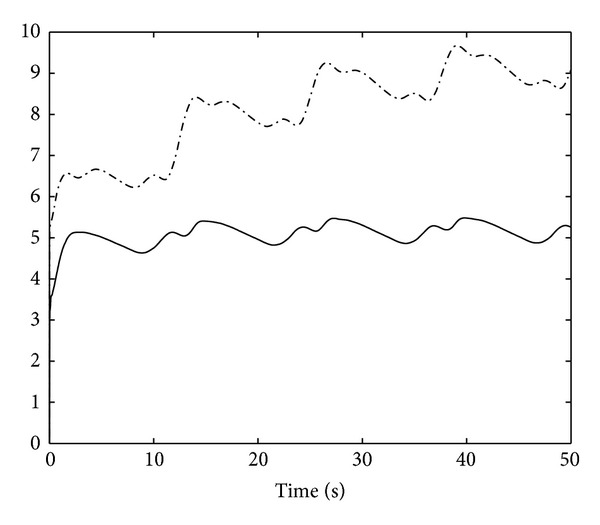
Adaptive parameters ${\hat{\alpha }}_{1}$ and ${\hat{\alpha }}_{2}$.

The simulation is run under the initial conditions *x*_*j*,*i*_*j*__(*ϑ*) = 0, −*τ*_0_ ≤ *ϑ* ≤ 0, *j* = 1,2, *i*_*j*_ = 1,2, and ${\left[{\hat{\alpha }}_{1}(0),{\hat{\alpha }}_{2}(0)\right]}^{T}={\left[0,0\right]}^{T}$. The result of control scheme is displayed in Figures 1–5. Figures 1 and 2 demonstrate the outputs of system and the reference signals. The responses of state variables *x*_1,2_ and *x*_2,2_ are shown in Figure 3. The control input signals *u*_1_ and *u*_2_ are illustrated in Figures 4 and 5which depict the boundedness of adaptive parameters ${\hat{\alpha }}_{1}$ and ${\hat{\alpha }}_{2}$.

## 6. Conclusion

For a class of perturbed nonlinear MIMO time-varying delay systems in a block-triangular form, an adaptive neural control design is presented. Although there are some fluctuations of the systems and control output under the influence of interference, the required performance can be achieved in a short period of time by using the controller designed in this paper and guarantees the boundedness of all the signals in the closed-loop system. It is further extended on the bases in [14, 15], which makes it suitable for wider range of applications. The effectiveness of the proposed approach is provided by a simulation example.
